# Investigation of spatio-temporal cancer clusters using residential histories in a case–control study of non-Hodgkin lymphoma in the United States

**DOI:** 10.1186/s12940-015-0034-7

**Published:** 2015-06-05

**Authors:** Rikke Baastrup Nordsborg, Chantel D. Sloan, Haseeb Shahid, Geoffrey M. Jacquez, Anneclaire J. De Roos, James R. Cerhan, Wendy Cozen, Richard Severson, Mary H. Ward, Lindsay Morton, Ole Raaschou-Nielsen, Jaymie R. Meliker

**Affiliations:** Danish Cancer Society Research Center, Copenhagen, Denmark; Department of Preventive Medicine, Stony Brook University, Stony Brook, NY USA; Department of Health Science, Brigham Young University, Provo, UT USA; Department of Applied Mathematics, Stony Brook University, Stony Brook, NY USA; BioMedware, Inc, Ann Arbor, MI USA; State University of New York at Buffalo, Buffalo, NY USA; Department of Environmental & Occupational Health, Drexel University School of Public Health, Philadelphia, PA USA; Mayo Clinic College of Medicine, Rochester, MN USA; Department of Preventive Medicine and Pathology, and Norris Comprehensive Cancer Center, USC Keck School of Medicine, University of Southern California, Los Angeles, CA USA; Department of Family Medicine and Public Health Sciences, Wayne State University, Detroit, MI USA; Division of Cancer Epidemiology and Genetics, National Cancer Institute, Bethesda, MD USA; Program in Public Health, Stony Brook University, Stony Brook, NY USA

**Keywords:** Non-Hodgkin lymphoma, Cancer, Spatio-temporal cluster analysis, Residential histories, *Q*-statistics

## Abstract

**Background:**

Non-Hodgkin lymphoma (NHL) is an enigmatic disease with few known risk factors. Spatio-temporal epidemiologic analyses have the potential to reveal patterns that may give clues to new risk factors worthy of investigation. We sought to investigate clusters of NHL through space and time based on life course residential histories.

**Methods:**

We used residential histories from a population-based NHL case–control study of 1300 cases and 1044 controls with recruitment centers in Iowa, Detroit, Seattle, and Los Angeles, and diagnosed in 1998–2000. Novel methods for cluster detection allowing for residential mobility, called *Q*-statistics, were used to quantify nearest neighbor relationships through space and time over the life course to identify cancer clusters. Analyses were performed on all cases together and on two subgroups of NHL: Diffuse large B-cell lymphoma and follicular lymphoma. These more homogenous subgroups of cases might have a more common etiology that could potentially be detected in cluster analysis. Based on simulation studies designed to help account for multiple testing across space and through time, we required at least four significant cases nearby one another to declare a region a potential cluster, along with confirmatory analyses using spatial-only scanning windows (SaTScan).

**Results:**

Evidence of a small cluster in southeastern Oakland County, MI was suggested using residences 10–18 years prior to diagnosis, and confirmed by SaTScan in a time-slice analysis 20 years prior to diagnosis, when all cases were included in the analysis. Consistent evidence of clusters was not seen in the two histologic subgroups.

**Conclusions:**

Suggestive evidence of a small space-time cluster in southeastern Oakland County, MI was detected in this NHL case–control study in the USA.

## Background

Worldwide, there are more than 380,000 cases of non-Hodgkin lymphoma (NHL) diagnosed each year, accounting for approximately 2.7 % of all cancers [1]. In the second half of the 20th century the incidence rate of NHL rose rapidly, nearly doubling in many regions of the world [2]. The causes of this increase remain puzzling, though the time trend suggests the action of new causal agents that might be avoidable [3]. Known risk factors for NHL, including HIV/AIDS, immune dysfunction/suppression e.g. resulting from organ transplantation or autoimmune diseases, and family history of NHL [4–7], account for only a small proportion of the total NHL cases, prompting the need to identify new risk factors.

Spatial epidemiologic analyses have the potential to reveal patterns that point to new risk factors worthy of investigation. Spatial investigations of individual-level data, however, typically only examine location at time of diagnosis, despite the potential for long latency periods for cancers such as NHL. This is a result of limitations in spatial statistics, which almost always assume that individuals are immobile (e.g., [8–15]). New techniques, however, have recently been developed that allow for investigating spatial patterns in residential histories [16–18], and enable the analyst to identify if, where, and when clusters are present.

Our group has been involved in development of *Q*-statistics for evaluating space-time clusters in residential histories of case–control data [16, 19]. The *Q*-statistics utilize nearest neighbor calculations to evaluate local and global clustering at any moment in the life course of the residential histories of cases relative to the residential histories of controls. Recent analyses [20] indicate acceptable performance for identifying simulated clusters, and provide a framework for interpreting statistical significance in light of multiple testing through space and time. Here we apply *Q*-statistics to investigate if, where, and when cases cluster using residential histories from a multi-center NHL case–control study in four regions of the United States served by Surveillance, Epidemiology, and End Results (SEER) registries [21]. Prior work with this dataset has examined spatial clusters at pre-specified time slices 0, 5, 10, 15, and 20 years prior to diagnosis [22], but such approach does not fully account for the underlying temporal changes in place of residence and does not take into account that some residential addresses may be of longer duration than others. Herein we present a systematic examination of space-time clusters that accounts for the complete residential mobility of study participants in the National Cancer Institute (NCI)-SEER NHL study.

## Methods

### NHL case–control dataset

The study design has previously been described [21–24]. Briefly, we conducted a population-based case–control study of NHL in four U.S. areas served by NCI-SEER registries: the Detroit metropolitan area (Macomb, Oakland, and Wayne Counties), King and Snohomish counties in northwestern Washington State, the state of Iowa, and Los Angeles (L.A.) County [21]. Cases were defined as those with a diagnosis of NHL between 20 and 74 years old recruited between July 1, 1998 and June 30, 2000. In Iowa and Seattle, all consecutive cases were chosen. In L.A. and Detroit, all African American cases and a random sample of White cases were eligible for study, enabling oversampling of African American cases. Population controls with no previous diagnosis of NHL were recruited during the same time period and frequency matched to cases by age (within 5-year age groups), sex, race and SEER area. Random digit dialing was used to select controls under age 65, and older controls (≥65 years) were identified from Centers for Medicare and Medicaid Services eligibility files. Based on self-reported information, individuals with known HIV or AIDS were excluded from the study i.e., interviewers read a series of eligibility criteria that included “no known infection with HIV”, and participants had to confirm that they were eligible for the study. The response rates among eligible cases and controls contacted for interview were 76 % and 52 %, respectively. In total, 1321 cases and 1057 controls were included in the NCI-SEER study. Written informed consent and IRB approval were obtained by NCI and at each study center.

A residential calendar was sent to all participants prior to interview and they were ask to provide the complete address of each home they had lived in from date of birth to present, including the years they moved in and out. Residences included homes lived in for at least 6 consecutive months. Interviewers reviewed the residential calendars and asked responders about missing information. For the total 2378 participants, 21,530 residential addresses were reported, however 67 of these were excluded because they applied to time periods after date of diagnosis/recruitment. In total the study included 137,112 person-years from year of birth to year of diagnosis/reference year; controls were assigned a reference year comparable to the case diagnosis year. Residential addresses were geocoded using Geographic Data Technology’s MatchMaker SDK Professional Version 4.3. The latitude and longitude returned were based on the coordinate projection NAD83 and were set to an offset of approximately 8 meters (25 feet) from the centerline of the street segment [25]. Further, the geographical location of the residential address at time of interview was measured with Global Positioning System (GPS) outside the house and cross-checked with geographical coordinates from the automatic geocoding procedure [26]. The residences were automatically or interactively geocoded with minor operator assistance. Of the 137,112 person-years 73 % were geocoded with an acceptable precision level for this study (60 % matched at the house or street and 13 % matched at zip code centroid), while 19 % of the person-years were too imprecise (matched at the nearest ‘populated place’, county or state centroid), hence these residential addresses were excluded from the analyses. Lastly, 8 % of the person-years were not geocoded or had missing address information. On average, 80 % of each individual’s person-years were with acceptable precision, with an interquartile range of 57–94 %. Geocoding efficiency was almost similar among cases and controls and decreased in areas outside of the study states due to more frequent missing street information for older addresses. There was exact geocoding match for 30.1 % of the addresses of cases against 29.2 % of the addresses of controls. After exclusion of residential addresses with no or imprecise geocodes and participants with missing values for adjustment variables 1300 cases and 1044 controls remained for the spatial-temporal analyses. Among cases there were 408 diffuse large B-cell lymphomas and 315 follicular lymphomas.

### *Q*-statistics

Case–control cluster tests known as *Q*-statistics were developed for use with residential histories [16]. These have been described multiple times [19, 20, 27] but will be briefly reviewed here. Cluster detection analyses using *Q*-statistics were run using SpaceStat (BioMedware, Ann Arbor, MI).

*Q*-statistics are a type of nearest-neighbor method employed across space and time. The Cuzick-Edwards statistic forms the basic framework [9] that is a matrix of distances and case–control statuses that are used to assign each individual a sum of the number of cases within their *k* nearest neighbors. (The number of nearest neighbors allowed, *k*, is specified by the user). The most basic statistic is *Q*_*i*,*t*_^(*k*)^, which is a sum at a single time point of the number of *k* nearest neighbors of individual *i* that are cases and not controls. This statistic is mathematically represented as follows:

1 $$ {Q}_{i,t}^{(k)}\kern0.5em =\kern0.5em {c}_i{\displaystyle \sum_{j=1}^k{\eta}_{i,j,t}^{(k)}{c}_j} $$

Where for individuals *i* and *j*, *c*_*i*_ and *c*_*j*_ are defined to be 1 if and only if a case, and 0 otherwise. The term *η*_*i*,*j*,*t*_^(*k*)^ is a binary spatial proximity metric that is 1 when participant *j* is a *k* nearest neighbor at time *t* of participant *i*; otherwise it is 0. *Q*_*i*,*t*_^(*k*)^ may take on a range of values from 0 to *k* based on the fact that an individual can have up to *k* unique nearest neighbors. Every time a participant changes residences the statistic is recalculated.

Additional variations of the *Q*-statistics are also calculated:

2 $$ {Q}_i^{(k)}\kern0.5em =\kern0.5em {\displaystyle \underset{t={t}_0}{\overset{T}{\int }}{Q}_{i,t}^{(k)}}\kern0.5em dt $$

3 $$ {Q}_i^{(k)}\kern0.5em =\kern0.5em {\displaystyle \sum_{i=1}^{n_1}{Q}_{i,t}^{(k)}} $$

4 $$ {Q}^{(k)}\kern0.5em =\kern0.5em {\displaystyle \sum_{i=1}^{n_1}{Q}_i^{(k)}} $$

Equations 2 and 3 build on *Q*_*i*,*t*_^(*k*)^ to identify which cases are centers of spatial clusters through time (Equation 2), and whether there is global clustering at a particular time (Equation 3). Equation 3 is calculated by summing Equation 1 over all cases at that moment in time. By summing equation 2 over all cases, Equation 4 gives a measure of global case clustering of residential histories throughout the study area and over the entire study time period.

### Regression and covariate adjustment

Geographic variation in already known risk factors and covariates may cause clusters, however to detect clusters that exist beyond known risk factors and covariates, *Q*-statistics must allow for adjustment. This is done by replacing the null hypothesis of complete spatial randomness, with a null hypothesis that accounts for each individual’s probability of being a case based on his/her known risk factors and covariates. Thus, observed clusters would not be attributable to geographic variation in the modeled risk factors and covariates, but instead would be due to geographic pattern in some other, perhaps unknown, risk factor. For this study several covariates were included. Logistic regression was conducted using SAS (9.3) for each variable mutually adjusted for the other variables in the model, which were age of referral, study center, sex, education, race and whether the home was treated for termites before 1988. All covariates except for age were coded as categorical variables. According to the logistic regression equation 1/1 + e^-z^, where z = B_0_ + B_1_x_1_…B_n_x_n_, the coefficients for each variable along with values for each individual were used to assign individual probabilities in the adjusted analysis. Covariates used for adjustment are listed for cases and controls in Table 1. Adjustment values fell between 0.33 and 0.78.

**Table 1:** Descriptive statistics for NHL cases and controls

Covariates	Cases *n* = 1300 (%)	Controls *n* = 1044 (%)
Median age at referral (5–95 % percentile)	58 (33–73)	61 (33–73)
Study center		
Detroit	319 (24)	214 (20)
Iowa	361 (27)	276 (26)
Los Angeles	319 (24)	273 (26)
Seattle	322 (24)	294 (28)
Sex		
Male	711 (54)	546 (52)
Female	610 (46)	511 (48)
Education		
Low (<12 years)	128 (10)	111 (11)
Middle (12–15 years)	815 (62)	616 (58)
High (+16)	377 (29)	330 (31)
Race		
African American	110 (8)	151 (14)
White	1123 (85)	843 (80)
Other/unknown	88 (7)	63 (6)
Termite treatment in home		
Home not treated	853 (65)	717 (68)
None or don’t know if treated	244 (18)	176 (17)
At least one home treated before 1988	220 (17)	162 (15)

### Cluster analyses

*Q-*statistics were calculated on the dataset with and without statistical adjustment. *Q*-statistics for *k* = 15 nearest neighbors were calculated for each case, based on previous results that indicated 15 as an appropriate *k* for these data [20]. Analyses were performed on the total dataset (n_cases_ = 1300) and subset analyses were conducted among the most common histologic subtypes of NHL: Diffuse large B cell (*n* = 408) and follicular lymphoma (*n* = 315). The entire control population (*n* = 1044) was used as a comparison group in all analyses. Analogous to age-period-cohort models, our space-time analyses were conducted using three temporal measures: Locations mapped by calendar year, years prior to diagnosis/recruitment, and by age. Both unadjusted and adjusted analyses were run using each temporal measure, for all cases, follicular and diffuse NHL subtypes, resulting in a total of 18 runs of the program. We used 999 permutations to determine significance of each test.

Because of the large number of statistical tests conducted in *Q*-statistics run across complete residential histories, multiple testing bias is a concern. In simulation analyses designed to help account for multiple testing across space and through time we created clusters and evaluated the predictive capability of different versions of the local and global *Q*-statistics. Results of our simulation studies indicated at least four significant cases (*Q*_*i*_^(*k*)^*p* = 0.001 and *Q*_*i*,*t*_^(*k*)^*p* ≤ 0.05) nearby one another to declare a region a potential cluster, accompanied by confirmatory analyses using spatial-only scanning windows (SaTScan).

Additional cluster analyses using Kulldorf’s scan statistic [28] in SaTScan (v 9.0.1) were carried out on sub-sets of the original space-time data, which included the residential addresses of cases and controls in the year 1988 and 20 years prior to diagnosis. This was done because our results suggested a possible cluster in the period 1979–1996 (middle year 1988), and because a previous cluster investigation of the NCI-SEER case–control data had found areas of elevated NHL risk when a 20 year lag time was considered [22]. In SaTScan we used the Bernoulli model, a circular scan window and allowed a maximum cluster size of 25 % of the population at risk.

## Results

Results of unadjusted and adjusted cluster analyses with *Q*-statistics are shown in Table 2.

**Table 2:** Results of unadjusted and adjusted space-time cluster analyses by three different time scales

	^a^Global *Q* ^(*k*)^, *p*-value	^b^ *Q*_*i*_^(*k*)^, *p* = 0.001	^c^ *Q*_*i,t*_^(*k*)^, *p* ≤ 0.05	^d^Area, time period
Unadjusted				
All (*n* = 1300)				
Calendar year	8.31, 0.53	2	2	Oakland, MI, 1979-1996
Years prior to diagnosis	8.31, 0.53	4	3	Oakland, MI, 10-18
			1	Appanoose, IA, 13-22
Age	8.37, 0.28	0	-	-
Follicular (*n* = 315)				
Calendar year	3.45, 0.56	0	-	-
Years prior to diagnosis	3.45, 0.58	0	-	-
Age	3.48, 0.45	0	-	-
Diffuse (*n* = 408)				
Calendar year	4.12, 0.80	0	-	-
Years prior to diagnosis	4.11, 0.84	1	1	Oakland, MI, 10-20
Age	4.16, 0.71	0	-	-
Adjusted^e^				
All (*n* = 1300)				
Calendar year	8.31, 0.31	2	2	Oakland, MI, 1979-1998
Years prior to diagnosis	8.31, 0.31	2	2	Oakland, MI, 1-18
Age	8.37, 0.13	1	1	Oakland, MI, 28–35, 41-48
Follicular (*n* = 315)				
Calendar year	3.45, 0.42	0	-	-
Years prior to diagnosis	3.45, 0.42	1	1	Oakland, MI, 5-20
Age	3.48, 0.32	0	-	-
Diffuse (*n* = 408)				
Calendar year	4.12, 0.47	1	1	Oakland, MI, 1972-1998
Years prior to diagnosis	4.11, 0.48	2	2	Oakland, MI, 9-20
Age	4.16, 0.37	0	-	-

Analyses were performed with *Q*-statistics, based on 15 nearest neighbours and 999 permutations. For each analysis the table lists ^a^the global *Q*-statistic and its *p*-value, ^b^the number of cases with a *Q*_*i*_^(*k*)^, *p*-value of 0.001 and among these ^c^ the number of cases that are co-located and with *Q*_*i*,*t*_^(*k*)^, *p*-values ≤ 0.05 and ^d^the geographic area and time of the cluster. ^e^Adjusted for age, sex, education, home termite treatment, race and site of recruitment. All controls (*n* = 1044) were use in all analyses

The global *Q*-statistic was not statistically significant for any of the analyses, indicating that the overall distribution of cases and controls was not clustered; however local clusters can exist even when global clustering is not detected [29].

A small cluster was identified in the southeastern part of Oakland County, MI near Detroit in several of the analyses both before and after adjustment (with *Q*_*i*_^(*k*)^, *p* = 0.001 and *Q*_*i*,*t*_^(*k*)^, *p* ≤ 0.05). The largest cluster had three cases at its center and was found using the total dataset from 10 to 18 years prior to diagnosis. A single case of NHL was also identified as a center of a cluster in Southern Iowa 13–24 years prior to diagnosis in the unadjusted analysis of all cases. However, none of the identified clusters had enough significant cases (four) to be declared a true cluster according to our simulation study [20]. Additional analyses with scan statistics (SaTScan) based on the residential locations of cases and controls in 1988 did not identify any significant clusters of NHL (most likely cluster was in Oakland County, MI, *p* = 0.33). But when the residential locations 20 years prior to diagnosis/recruitment were considered, a statistically significant cluster of 19 cases was detected in southeast Oakland County, MI near Detroit with *p* = 0.041, and a second and borderline significant area of reduced NHL risk was detected in central Los Angeles, CA with *p* = 0.052. Separate analyses of NHL subgroups (follicular and diffuse) only identified clusters of one or two cases, thus they were regarded as insignificant findings.

## Discussion

Based on lifetime residential histories this multicenter case–control study identified a borderline small cluster of NHL in southeastern Oakland County 10–18 years prior to diagnosis and in the 1980s and 1990s both before and after adjustment for age, sex, education, home termite treatment, race and site of recruitment. The cluster was confirmed by spatial-only scan analysis based on residential locations of cases and controls 20 years prior to diagnosis/recruitment.

NHL is a heterogeneous group of malignancies and potential explanations for the borderline cluster found in the total dataset should be suggested with caution. Clusters of NHL of no specific subtype would imply a common etiology for the total group of NHL; however recent research indicates that risk factors differ between subtypes [30]. We tried to take this into account by performing separate analyses for the two most common subtypes of NHL, but we did not detect any clusters among these more homogeneous subgroups of NHL. This could be because there were no subtype-specific clusters present, but it is also possible that our samples became too small for clusters to be detected when we subdivided the study population. For example, residential histories of 408 cases of diffuse large B cell and 315 cases of follicular NHL become geographically dispersed when mapped across the entire U.S.

The NCI-SEER NHL case–control study included self-reported lifetime residential addresses and detailed information on a large number of potential risk factors related to NHL. These data allowed us to utilize a novel method that can account for residential mobility of cases and controls in the search for local space-time clusters and at the same time adjust for potential confounders. This has only been done in few previous spatial studies of NHL [22, 31]. Cases were recruited from population-based cancer registries and controls were selected by random-digit dialing, and frequency matched on age, gender, race and SEER area [26]. The geographical location of residential addresses of participants at time of interview was determined with a high degree of precision as they were measured with GPS outside the house and cross-checked with geographical coordinates from the automatic geocoding procedure [26]. However, location of past residential addresses was less precise because reporting of older addresses was more often incomplete. The study had low participation rates both among cases (76 %) and controls (52 %) [21], and selection bias could have occurred. In epidemiologic surveys it is common to have higher response rates among cases than among controls, and persons with high socioeconomic status (SES) are more likely to participate than persons with lower SES. A previous investigation of differences between respondents and nonrespondents in the NCI-SEER case–control study showed that at census level respondents had higher SES than nonrespondents [24], but due to lack of data it was not possible to evaluate individual level differences.

Controls below 65 years of age were selected by random digit dialing, however this method does not guarantee unbiased selection of controls [32], and it is possible that the proportion of affluent persons is higher among the responding controls compared to the general population due to differential response. If responding controls also tend to live in certain neighborhoods, selection bias could eventually hide clusters of NHL in wealthy areas and maybe also lead to detection of false positive clusters in deprived areas. Further, bias could also have occurred if non-participating controls tended to live in certain areas e.g. near heavy industry, power plants or other areas of potential exposure. Consequently, “exposed” residential addresses among controls would be underreported and could also lead to identification of false positive clusters. In an effort to investigate geographic selection bias in this study population, a previous study investigated area-level demographic and socioeconomic differences between participants and nonparticipants, but found that differences in income and education among participants and nonparticipants did not have a large impact on the risk of NHL [24]. Further, the study showed that after adjusting for covariates there was no geographic clustering of non-responders suggesting limited potential bias in geographic analyses.

Potential recall bias is also a concern in the present study, as it may have caused differential misclassification of the covariates used for adjustment. However, results of the crude and the adjusted cluster analyses were fairly similar. Residential addresses were self-reported, and although the interviewers tried to ensure that the residential calendars were as complete as possible, recall bias cannot be entirely excluded, as cases could have reported their residential addresses more precisely than controls. The proportion of addresses with an exact geocoding match was slightly higher for cases (30.1 %) than controls (29.2 %), therefore the location of residential addresses could be slightly biased, but it is difficult to assess if this has influenced results of the cluster analysis.

In a previous spatial cluster study of the NCI-SEER NHL residential data that relied on time slices, Wheeler et al. used Generalized Additive Models (GAM) to detect areas of elevated risk of NHL in Detroit and Iowa at several points in time and areas of both elevated and reduced risk in Los Angeles at a lag time of 20 years [22]. These results were obtained after adjustment for the same covariates as in the present study. *Q*-statistics in the present study provided some evidence in support of the cluster near Detroit, and also detected a single case as center of a cluster in Southern Iowa but did not identify clusters in Los Angeles. Given the possibility of multiple testing bias resulting from the large number of space-time statistical analyses in *Q*-statistics, our requirement of four cases nearby one another and persistent across the life course was not reached. However, our confirmatory analyses conducted with the spatial scan statistic (SaTScan), identified a statistically significant cluster of elevated risk in southeast Oakland County, MI and a borderline significant area of reduced risk in central Los Angeles 20 years prior to diagnosis.

The similarities between the findings of the former [22] and the present study are notable because of several differences between the two studies. First, there are methodological differences, because the *Q*-statistics evaluate clusters over the entire life course of cases and controls, and thus allow us to identify clusters that persist over time, while the spatial GAM approach used in the former study required relevant lag time-slices to be specified prior to the spatial analysis [22]. Therefore, the identification of areas of elevated risk with the GAM method cannot fully account for the residential mobility of the study population. Second, the present study included all study participants with reasonably well geocoded residential addresses (1300 cases and 1044 controls) , while the former only included participants who had continuously lived in one of the SEER recruitment areas for 20 years prior to diagnosis/reference date, corresponding to 842 cases and 680 controls [22]. Further, the former study conducted analyses separately in each study site, whereas we chose to conduct the analyses on the entire dataset because of mobility across study sites and within the United States over the continuous life course. Therefore, the finding of a small cluster of elevated risk near Oakland County, MI by *Q*-statistics, spatial GAM, and SaTScan lends credence to the possibility that the cluster may be real.

*Q*-statistics have previously been used to investigate potential clusters of residential addresses of NHL cases and controls in a Danish nationwide study. That study was entirely register-based and included 3210 cases and two independent, randomly selected control groups of 3210 individuals each. A few small clusters of NHL were identified by both *Q*-statistics and SaTScan, but results were not consistent across the two control groups, and when analyses were repeated with the two control groups combined, no clusters were detected. Therefore, clusters found with each of the two control groups seemed to be driven by the distribution of controls rather than the cases, thus they were regarded chance findings [31]. In the present study *Q*-statistics and SaTScan suggested a single cluster of NHL in southeastern Oakland County, MI, but we did not have the possibility to test the validity of the finding with a second control group, thereby tempering our confidence in the resulting cluster.

Consistent with the previous Danish study the present study did not identify any convincing cluster of diffuse large B cell lymphoma or follicular lymphoma, however for both studies the subgroups were relatively small, and the ability of *Q*-statistics to detect potential clusters in datasets smaller than 500 cases and 500 controls has not been tested [20]. In the simulation study [20] we created clusters using the NCI-SEER NHL case–control residential data, which resulted in a guideline requiring four cases significant over the life course (*Q*_*i*,*t*_^(*k*)^, *p* = 0.001) and nearby one another (*Q*_*i*,*t*_^(*k*)^, *p* ≤ 0.05) to be able to distinguish a true simulated cluster from a false positive. Use of the same residential history dataset as in the simulation study should add strength to the present study, although perhaps our guideline was too restrictive given that the cluster in southeastern Oakland County, MI with just three significant *Q*-cases was confirmed by SaTScan (*p* = 0.041). This is ground for future work.

With only one suggestive cluster identified out of all the cases in the study from four different study regions the present study indicates that clusters of NHL are likely rare, but they can occur, maybe due to rare types of industrial point exposures, or perhaps due to concomitant exposure to an infectious agent. Given that HIV increases the risk of NHL, and that other infections may also play a role in the development of NHL, at least in some of the subtypes, it seems possible that a local virus could result in NHL clusters. However; if there is a long lag time between infection and NHL development the methods of the present study would only identify such a cluster if the infected NHL cases had lived close together for a substantial period of time. It also is possible that a rare industrial exposure permeated the cluster area approximately 10–20 years prior to diagnosis, but this is ground for future study. However, given the multiple tests that were conducted, it is also possible that the finding is due to chance alone.

## Conclusions

No global clustering was found in this NHL case–control study in the USA, but suggestive evidence of a small space-time cluster in southeastern Oakland County, MI approximately 10–20 years prior to diagnosis was detected. Use of a single control group tempers our enthusiasm that the cluster is real although possible explanatory factors merit exploration.
